# Early predictive value of prediction models for mortality after transcatheter aortic valve replacement: a systematic review and meta-analysis

**DOI:** 10.3389/fcvm.2026.1757852

**Published:** 2026-07-15

**Authors:** Ruiyan Wang, Mengyu He, Ziting Yuan, Jing Zhou, Lili Han, Xu Cheng, Yiming Chen, Feng Wang

**Affiliations:** 1 School of Nursing, Bengbu Medical University, Bengbu, Anhui, China; 2 Department of Cardiology, The First Affiliated Hospital of Bengbu Medical University, Bengbu, Anhui, China; 3 Nursing Department, The First Affiliated Hospital of Bengbu Medical University, Bengbu, Anhui, China; 4 Department of Hematology, The First Affiliated Hospital of Bengbu Medical University, Bengbu, Anhui, China; 5 Department of Internal Medicine, The First Affiliated Hospital of Bengbu Medical University, Bengbu, Anhui, China

**Keywords:** machine learning model, mortality risk, prediction model, risk stratification, TAVR

## Abstract

**Background:**

Transcatheter aortic valve replacement (TAVR) is increasingly used due to the rising incidence of aortic stenosis (AS). Early identification of mortality risk after TAVR is challenging. Although various prediction models have been developed, no systematic review has evaluated their effectiveness in predicting mortality risk. Therefore, this study aimed to systematically evaluate the performance of models for early prediction of mortality risk after TAVR, so as to provide evidence-based support for the future development or updating of risk assessment tools.

**Methods:**

Databases (PubMed, Web of Science, Embase, and Cochrane Library) were systematically searched for studies on tools for predicting the risk of mortality after TAVR, up to June 2024. PROBAST was used to assess the risk of bias in the included studies. A subgroup analysis was conducted based on different time points.

**Results:**

This systematic review included 36 studies with 272,390 patients receiving TAVR and 6 major scoring tools encompassing 23 new machine learning models. The meta-analysis showed that the concordance index (C-index) was 0.610 (95% CI: 0.588–0.632) for European System for Cardiac Operative Risk Evaluation I (EuroSCORE I), 0.615 (95% CI: 0.588–0.643) for EuroSCORE II, 0.578 (95% CI: 0.531–0.625) for French Aortic National CoreValve and Edwards II (France II), 0.594 (95% CI: 0.554–0.633) for the OBSERVANT score, 0.648 (95% CI: 0.622–0.674) for the Society of Thoracic Surgeons (STS) risk model, 0.632 (95% CI: 0.616–0.648) for the American College of Cardiology Transcatheter Valve Therapy (ACC TVT) risk model, and 0.705 (95% CI: 0.677–0.733) for summarized machine learning models.

**Conclusion:**

Determining the predictive performance of current established risk assessment tools for predicting the risk of modality after TAVR is challenging. Machine learning models seem to be more effective. Therefore, future research should include more subjects to develop more accurate models.

**Systematic Review Registration:**

https://www.crd.york.ac.uk/PROSPERO/, identifier CRD42023485237.

## 1 Introduction

The prevalence of moderate to severe valve disease in the US is 2.5% in the geriatric population and up to 13.3% in people older than 75 years. In Europe, the prevalence of aortic stenosis (AS) is 0.4% in the overall population and up to 2%–3% in the geriatric population (1, 2). Transcatheter aortic valve replacement (TAVR) is a minimally invasive alternative to surgical aortic valve replacement (SAVR). As TAVR is increasingly used, the annual use of TAVR in the US has exceeded all forms of SAVR. Significant improvements in quality of life (QOL) can be observed at 30 days after TAVR, while no obvious improvement in QOL is seen at 30 days after SAVR due to its greater surgical trauma (3). Advanced age, comorbidities and frailty may affect the beneficial impact of interventions on quality of life, and available tools for assessing QOL in patients with aortic stenosis have also been described in studies (4). However, the mortality rate after TAVR is increasing due to complications, suitability of valves and other reasons (5).

Currently, in addition to risk prediction models, echocardiographic parameters and biomarkers can also be used to predict the risk of mortality after TAVR, but their predictive utility remains limited in routine clinical settings (6). Several tools have been developed to predict the risk of mortality. For example, the European System for Cardiac Operative Risk Evaluation I (EuroSCORE I), based on one of the largest, most complete and accurate databases in European cardiac surgical history, was developed in 1999 (7) and upgraded to EuroSCORE II in 2012 by updating the database and refining some of the risk factors such as renal dysfunction (8). There are also well-established and stable risk prediction models, such as the French Aortic National CoreValve and Edwards II (France II), the American College of Cardiology Transcatheter Valve Therapy (ACC TVT) risk model, and the Society of Thoracic Surgeons (STS) risk model. However, the performance of these models remains elusive. In recent years, with their popularization and application in clinical settings, machine learning models for predicting mortality risk after TAVR have been developed.

However, currently, there is still no systematic evidence for the predictive value of these models. Therefore, this study aimed to systematically review the value of models for predicting early mortality risk after TAVR, so as to provide evidence for promoting the development and updating of simple scoring tools for predicting early mortality risk after TAVR.

## 2 Methods

This study was conducted based on PRISMA 2020 guidelines and registered on PROSPERO (ID: CRD42023485237).

### 2.1 Search strategy

PubMed, Cochrane, Embase, and Web of Science were systematically searched until June 20, 2024. Search terms were designed by combining subject headings and free-text terms. There was no restriction on regions and the year of publication. The search strategy is detailed in Supplementary Material 1.

### 2.2 Study eligibility criteria

#### 2.2.1 Inclusion criteria

1. The included study subjects were patients receiving TAVR.
2. The types of studies included were case-control studies, cohort studies, nested case-control studies, and case-cohort studies.
3. Studies constructing complete models for predicting mortality risk after TAVR.
4. Studies validating previous predictive models or scoring tools.
5. Included studies were published in English.

#### 2.2.2 Exclusion criteria

(1) Meta-analyses, reviews, guidelines, and expert consensuses; (2) studies that only analyzed risk factors, without developing complete machine learning models; (3) Studies that did not report any of the following outcome measures: ROC curve, c-statistic, concordance index (C-index), sensitivity (SEN), specificity (SPE), accuracy, recovery rate, accuracy rate, confusion matrix, diagnostic fourfold table, F1 score, and calibration curve.

### 2.3 Data extraction procedures

The searched studies were imported into EndNote to remove duplicates. Then, their titles and abstracts were read to exclude irrelevant studies. Next, the full texts of the remaining studies were reviewed to determine eligible studies. Data were extracted using a standard data extraction spreadsheet, including title, first author, study type, patient source, follow-up time, number of deaths, total number of cases, number of deaths in the training set, total number of cases in the training set, generation method used in the validation set, over-fitting method, number of deaths in the validation set, number of cases in the validation set, method for handling missing values, variable screening/feature selection method, model type, and modeling variables.

Extracted data were cross-checked. Disagreements, if any, were addressed by a third investigator (FW).

### 2.4 Risk of bias assessment

The risk of bias (RoB) in the included studies was assessed using PROBAST. This tool contains the following 4 domains: participants, predictors, outcome, and analysis to assess the RoB and applicability. The four domains contain 2, 3, 6 and 9 specific questions, respectively, and each question is answered as yes (Y), probably yes (PY), probably no (PN), no (N), or no information (NI). A domain was considered to be at high RoB if at least one question was answered as “N”. The overall RoB was considered high if at least one domain was judged to be at a high RoB. The overall RoB was considered low if all domains were rated as having a low RoB. The overall RoB was considered unclear if an unclear RoB was noted in at least one domain and it was low for all other domains.

The included studies were evaluated separately by two investigators (MH, ZC) based on PROBAST. The results of RoB assessment were cross-checked by them. Disagreements, if any, were addressed by a third investigator (FW).

### 2.5 Statistic methods

Meta-analysis of C-index was performed to evaluate the accuracy of prediction models for mortality after TAVR. When the average C-index across all samples ranged from 20% to 80%, C-index values were not required to be logit-transformed. When it was less than 20% or more than 80%, the logit transformation was used. When there were a large number of values of 0% and/or 100%, the double-arcsine transformation was used. For some original studies that did not provide standard errors or confidence intervals, values were estimated using the method proposed by Debray TP et al. (9). Heterogeneity was quantified using *I*^2^. If *I*^2^ > 50%, a random-effects model was used; otherwise, a fixed-effects model was used. When excessive heterogeneity was found, sensitivity analysis, subgroup analysis, and meta-regression were used to explore the sources of heterogeneity. Meta-analysis was carried out using Stata 15.0 and R4.4.2.

## 3 Results

### 3.1 Study screening

**Figure 1:**
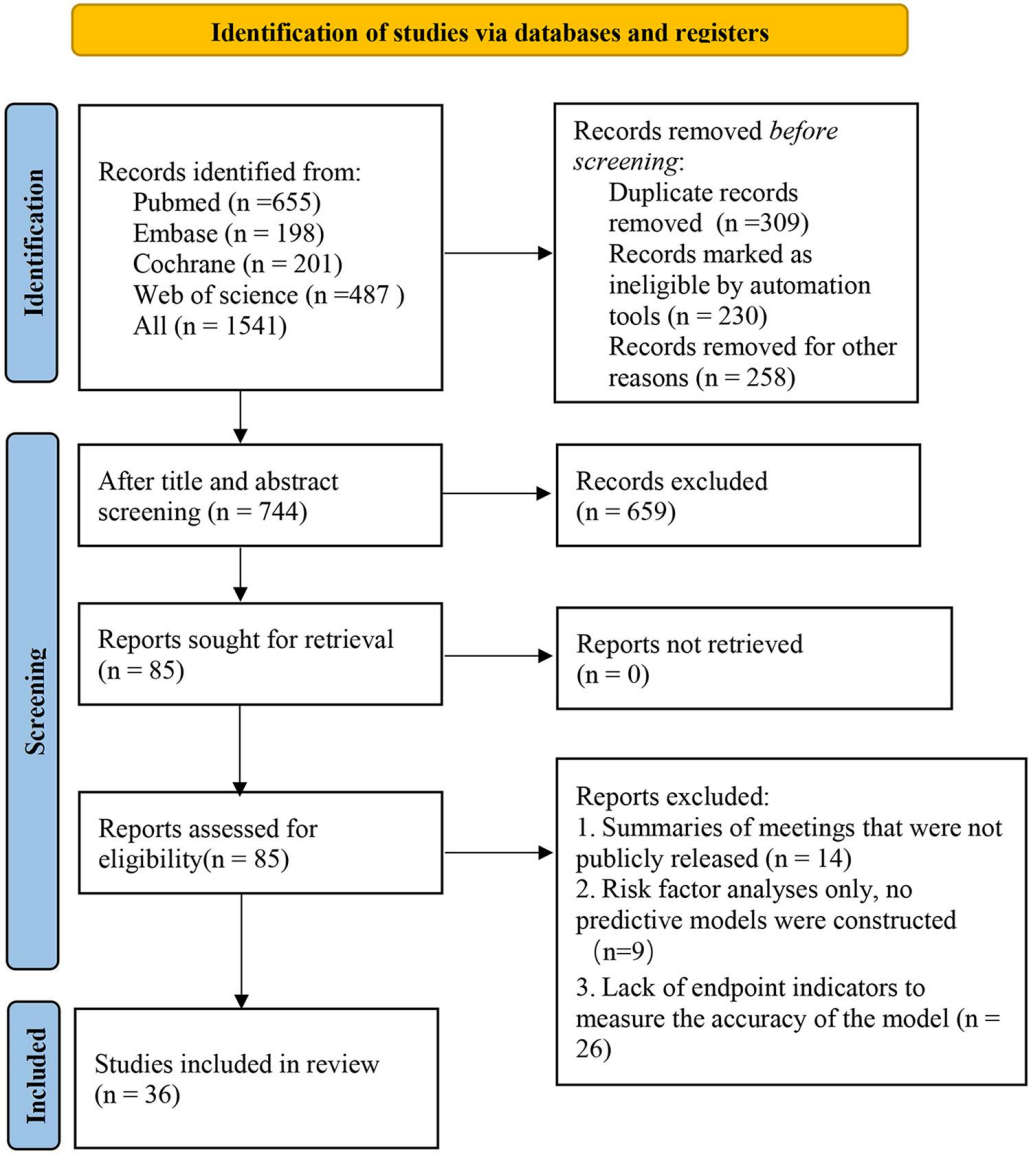
Study screening procedure.

1,641 studies were searched from the databases. Of them, 409 duplicates and 1,183 irrelevant studies were excluded after reading their titles and abstracts. The full texts of the remaining studies were downloaded and read. Then, 49 of them were excluded due to only analyzing risk factors without constructing prediction models. Finally, 36 studies (10–45) were included. The study screening procedure is detailed in Figure 1.

### 3.2 Characteristics of the included studies

The included studies involved 272,390 patients. Mortality at various time points after TAVR mainly included in-hospital mortality (25, 28, 34, 40), 30-day mortality (14, 16, 20, 26–28, 30, 32, 38, 40–42), 1-year mortality (10, 14, 21, 24, 26, 29, 46), 3-year mortality (31, 36), and 5-year mortality (35). These studies were published between 2011 and 2024. There were 6 well-established tools. Of them, 8 studies validated EuroSCORE I, 13 validated EuroSCORE II, 5 validated France II, 5 validated the OBSERVANT score, 10 validated the STS risk model, and 5 validated the ACC TAT risk model. Of the included studies, 9 were from the US, 3 from the UK, 3 from France, 5 from Germany, 2 from Italy, and 5 from the Netherlands. Among the included studies, there were 11 single-center studies, 16 multicenter studies, and 10 studies based on registration databases (Table 1).

**Table 1:** General characteristics of the included studies.

No	First author	Year of publication	Country of author	Study type	Patient source	Duration of follow-up for mortality	Number of deaths	Total number of cases	Total number of cases in the training set	Method in the validation set	Total number of cases in the validation set	Processing methods for missing values	Model type
1	Agasthi, P.	2021	America	Retrospective cohort study	Multicenter		148	1,055	1,055	5-fold cross-validation		Deletion	gradient boosting machine learning (GBM) model, TAVI2-SCORE, CoreValve Score
2	Ariyaratne, T. V.	2011	Australia	Retrospective cohort study	Registration database	30d		4,812	3,544	Prospective validation	1,268	Deletion	multiple logistic model (AVR-Score)
3	Yamamoto, M.	2021	Japan	Retrospective cohort study	Multicenter	1 year		2,575	1,931	Random sampling	644	Deletion	simple office models
4	Wang, T. K. M.	2015	New Zealand	Retrospective cohort study	Single center	3.8 ± 2.4 years	18	620		External validation	620		EuroSCORE, EuroSCORE II,STS score,Aus-AVR score)
5	Yatsynovich, Y.	2017	America	Retrospective cohort study	Single center	30d	10	182		External validation	182		TAVR-RS、STS PROM、EuroSCORE II
6	Mamprin, M.	2021	Netherlands	Retrospective cohort study	Single center	1 year	30	270	270	5-folded cross-validation		Minimum value	GBDT
7	Al-Farra, H.	2022	Netherlands	Retrospective cohort study	Registration database	30d	368	9,144	6,855	Prospective	2,289	Random	TAVI-NHR
8	Edwards, F. H.	2016	American	Retrospective cohort study	Registration database	Hospital stay	1,030	20,586	13 718	External validation	6,868	Median	TVT Registry model
9	Lantelme, P.	2019	France	Retrospective cohort study	Multicenter	1 year	267	1,736	1,425	External validation	311	No processing	CAPRI score
10	Chen, Y.	2023	UK	Retrospective cohort study	Multicenter		22	450	450	5-folded cross-validation			GBST
11	Al-Farra, H.	2020	Netherlands	Retrospective cohort study	Registration database	30d	280	6,177		External validation	6,177	Multiple imputation	STS, EuroSCORE-I, EuroSCORE-II, ACC-TAT, FRANCE-2, OBSERVANT, and German-AV
12	Lantelme, P.	2020	France	Retrospective cohort study	Registration database	1 year	3,702	20,443	10,221	Random sampling	10,222		Futile TAVI Simple (FTS) score
13	Martin, G. P.	2017	United Kingdom	Retrospective cohort study	Multicenter	30d	360	6,676		External validation	6,676	Multiple imputation	LES, ESII, STS,German AV, FRANCE-2,OBSERVANT, ACC TAT
14	Arnold, S. V.	2018	America	Retrospective cohort study	Multicenter	30d	1,025	21,661	15,163	Random sampling	6,498	Median	Logistic regression
15	Kwiecinski, J.	2023	Poland	Retrospective cohort study	Multicenter	1 year	163	1,427	604	External validation	823		XGBoost model.
16	Hernandez-Suarez, D. F.	2019	USA	Retrospective cohort study	Registration database	Hospital stay	390	10,883	7,615	Random sampling	3,268	Deletion	NIS TAVR score
17	Lertsanguansinchai, P.	2023	Thailand	Retrospective cohort study	Single center	30d and 1 year	30d:7	178	142	Random sampling	36	Deletion	the decision tree model
							1 year:24	178	142	Random sampling	36	Deletion	the decision tree model
18	Aziz, M.	2018	Canada	Retrospective cohort study	Multicenter	30d	78	1,550		External validation	1,550		EuroSCORE, EuroSCORE II, STS
19	Codner, P.	2018	USA	Retrospective cohort study	Single center	30d	Hospital stay：14	1,038		External validation	1,038		The ACC/TVT registry mortality risk score, the STS-PROM score and the EuroSCORE II
							30d：30						
20	Jung, C.	2022	Germany	Retrospective cohort study	Multicenter	1 year	3,610	24,686	20,704	External validation	3,982	Deletion	TAVR-Risk (TARI) model
21	Martin, G. P	2018	UK	Retrospective cohort study	Multicenter	30d	326	6,339	2,969	Bootstrap		Algorithm (Multiple imputation)	UK-TAVI CPM
22	de Terwangne, C.	2023	Belgium	Retrospective cohort study	Single center	2 year	89	345	345	5-folded cross-validation		Deletion	OLD-TAVR score
23	Silva, L. S.	2015	Brazil	Retrospective cohort study	Multicenter	30d	38	418		External validation	418		EuroSCORE I, EuroSCORE II, Society of Thoracic Surgeons (STS) score, Ambler score (AS) and Guaragna score (GS)
24	Hermiller, J. B.	2016	USA	Retrospective cohort study	Single center	30d	214	3,687	2,482	Random sampling	1,205		simple score
						1 year	840	3,687	2,482	Random sampling	1,205		simple score
25	Alhwiti, T.	2023	USA	Retrospective cohort study	Registration database	Hospital stay	1,113	54,739		External validation			
26	Penso, M.	2021	Italy	Retrospective cohort study	Single center	5 year	212	471	424	10-folded cross-validation	47	Deletion	LR
27	Beurton, A.	2021	France	Retrospective cohort study	Single center	3 year	168	1,101	771	Random sampling	330	Algorithm (Multiple imputation)	LR
28	Maeda, K.	2022	Japan	Retrospective cohort study	Registration database	1 year	1,316	17,655	12,316	Random sampling	5,339	No processing	J-TVT registry model
29	Kjonas, D	2021	Norway	Retrospective cohort study	Multicenter	30d	29	459	218	External validation	241	No processing	LR
30	Capodanno, D.	2014	Italy	Retrospective cohort study	Multicenter	30d	114	1,878	1,256	Random sampling	622		the OBSERVANT Score
31	Arsalan, M	2018	Germany	Retrospective cohort study	Registration database	30d	60	946		External validation	946	Unclear	STS/ACC TAT, EuroSCORE I, EuroSCORE II, STS-PROM, and German AV Score
31	Arsalan, M	2018	Germany	Retrospective cohort study	Registration database	Hospital stay	46	946		External validation	946		STS/ACC TAT
32	Lopes, R. R.	2023	Netherlands	Retrospective cohort study	Multicenter	30d	410	11,291	11,291	10-folded cross-validation		Multiple imputation	LR, XGBoost
33	Al-Farra, H.	2021	Netherlands	Retrospective cohort study	Registration database	30d	280	6,177	6,177	Bootstrap		Random	FRANCE-2, ACC-TAT
34	Francesco Pollari	2023	Switzerland	Retrospective cohort study	Single center	1 year	67	565	565	10-folded cross-validation		Deletion	RF
35	Andreas Leha	2023	Germany	Retrospective cohort study	Multicenter	30d	875	28,147	22,283	External validation	5,864		TRIMpre
36	Maria Zisiopoulou	2023	Germany	Retrospective cohort study	Multicenter	1 year	30	284		External validation		Deletion	LR

### 3.3 Risk of bias (RoB) assessment

**Figure 2:**
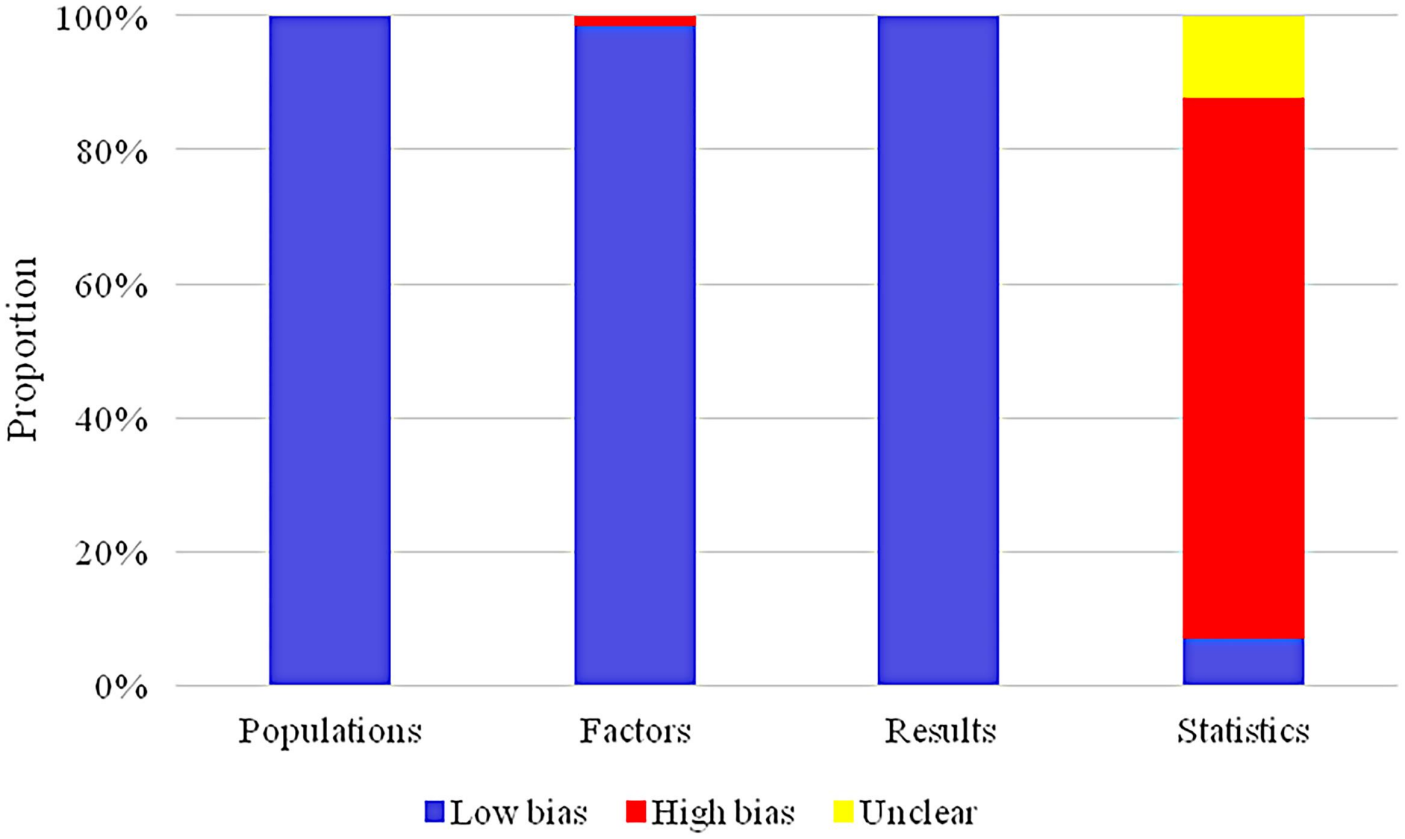
Results of risk of bias assessment of the included models.

The included studies were cohort studies involving 130 models. Therefore, all the studies had a low RoB in the patient selection. One study did not handle missing data, which may result in a high RoB. Regarding outcome measures, the rationality of their definitions was assessed, and the outcome of interest was mortality. Therefore, all the studies had a low RoB in outcomes. In statistical analyses, the principles of estimation of the number of cases were not satisfied in a large number of studies; missing values were not rationally handled; and goodness-of-fit was not assessed. Therefore, these studies were at high RoB from statistical analyses (Figure 2).

### 3.4 Mortality after TAVR

**Figure 3:**
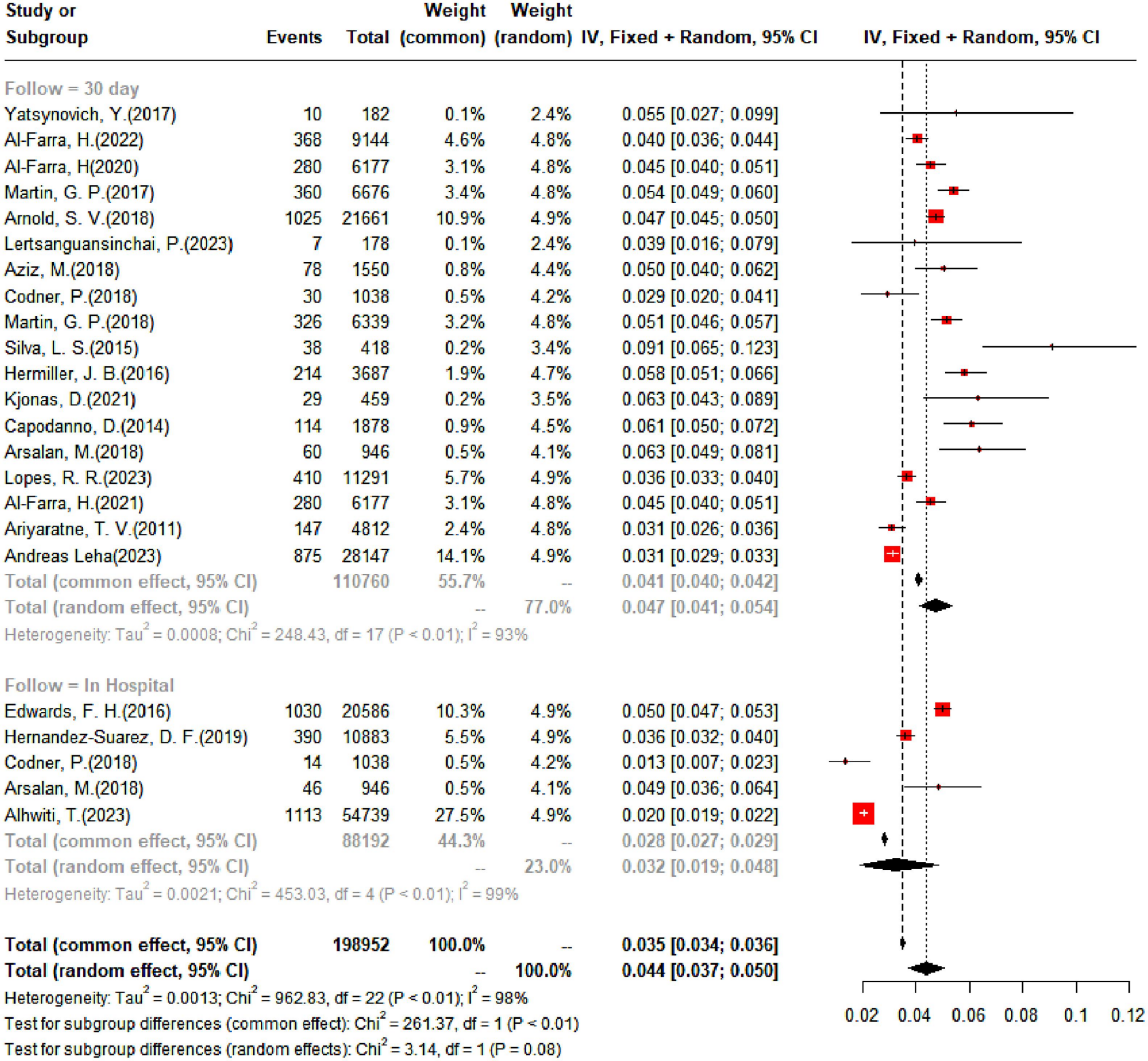
Forest plot for meta-analysis of short-term in-hospital mortality after TAVR.

**Figure 4:**
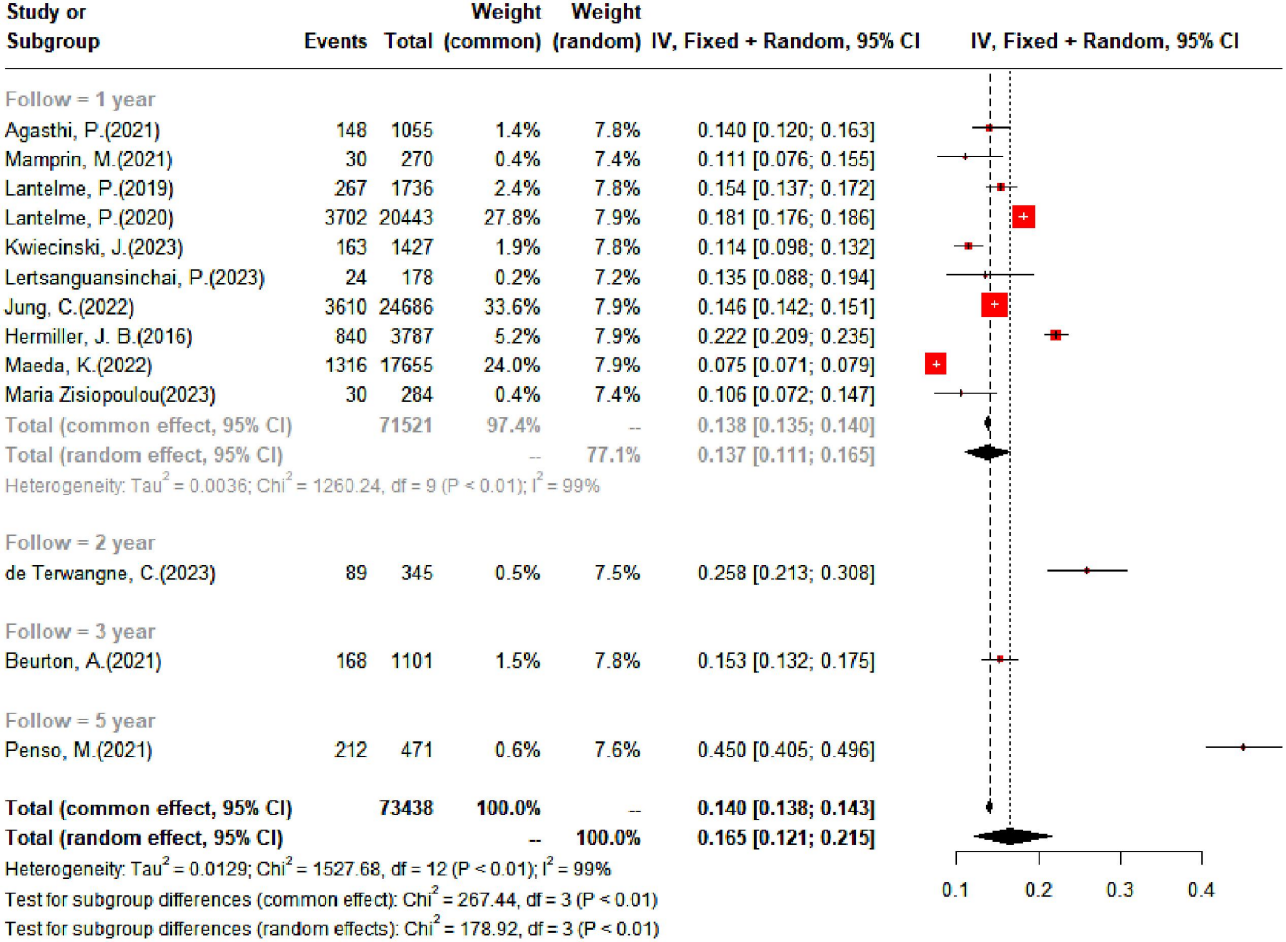
Forest plot for meta-analysis of long-term in-hospital mortality after TAVR.

Meta-analysis of mortality in all the included studies was performed. Short-term mortality was described in 23 studies. The results indicated that the in-hospital mortality was 3.2% (95% CI: 1.9%–4.8%, 5 studies), and the 30-day mortality was 4.7% (95% CI: 4.1%–5.4%, 18 studies, Figure 2). Long-term mortality was described in 13 studies. The 1-year mortality was 13.7% (95% CI: 11.1%–16.5%, 10 studies), and the mortality over 1 year was 16.5% (95% CI: 12.1%–21.5%, 3 studies, Figures 3, 4).

### 3.5 Models for predicting mortality after TAVR

Regarding various models for predicting mortality at different time points after TAVR, the fixed-effects model was used to pool data. Nine studies explored EuroSCORE I, and the C-index was 0.625 (95% CI: 0.594–0.656). EuroSCORE II was validated in fourteen studies, and the C-index was 0.621 (95% CI: 0.594–0.649). France II was validated in four studies, and the C-index was 0.578 (95% CI: 0.531–0.625). The OBSERVANT score was validated in four studies, and the C-index was 0.594 (95% CI: 0.554–0.633). The STS risk model was validated in twelve studies, and the C-index was 0.648 (95% CI: 0.622–0.674). The ACC TAT risk model was validated in five studies, and the C-index was 0.632 (95% CI: 0.616–0.648).

The funnel plot was used to analyze publication bias for assessment tools validated in more than 10 studies. The results showed that EuroSCORE I (Supplementary Figure S1), EuroSCORE II (Supplementary Figure S2), the STS risk model (Supplementary Figure S3), and newly developed machine learning models (Supplementary Figure S4) did not seem to have significant publication bias.

The sensitivity was 0.57 (95% CI: 0.54–0.60) for EuroSCORE I (validated in 5 studies), 0.60 (95% CI: 0.58–0.62) for EuroSCORE II (validated in 11 studies), 0.63 (95% CI: 0.57–0.68) for the STS risk model (validated in 10 studies), and 0.70 (95% CI: 0.57–0.80) for the ACC TAT risk model (validated in 3 studies). The specificity was 0.59 (95% CI: 0.55–0.63) for EuroSCORE I (validated in 5 studies), 0.58 (95% CI: 0.54–0.62) for EuroSCORE II (validated in 11 studies), 0.62 (95% CI: 0.57–0.67) for the STS risk model (validated in 10 studies), and 0.55 (95% CI: 0.48–0.62) for the ACC TAT risk model (validated in 3 studies), as detailed in Tables 2, 3.

**Table 2:** Various models for the prediction of mortality at different time points after TAVR.

Model	Follow-up	*n*	Events	Sample size	C-index (95% CI)	*I* ^2^
EuroSCORE I						
	In-hospital	NA	NA	NA	NA	NA
	30d	6	1,368	37,887	0.630 (0.579–0.681)	84.6
	1 year	3	3,998	30,025	0.598 (0.589–0.607)	0
	3 years	NA	NA	NA	NA	NA
	5 years	1	18	620	0.752 (0.652–0.852)	NA
	Overall	10	5,384	68,532	0.625 (0.594–0.656)	89.6
EuroSCORE II						
	In-hospital	1	14	1,038	0.746 (0.606–0.886)	NA
	30d	8	1,731	45,161	0.616 (0.567–0.666)	92.1
	1 year	3	1,971	11,328	0.618 (0.581–0.656)	40.2
	3 years	1	168	1,101	0.600 (0.555–0.645)	NA
	5 years	2	230	1,091	0.644 (0.538–0.751)	74.6
	Overall	15	4,114	59,719	0.621 (0.594–0.649)	86.1
France II						
	In-hospital					
	30d	4	926	19,172	0.575 (0.518–0.631)	89.3
	1 year	1	95	823	0.580 (0.530–0.630)	NA
	3 years	NA	NA	NA	NA	NA
	5 years	NA	NA	NA	NA	NA
	Overall	5	1,021	19,995	0.578 (0.531–0.625)	86.2
OBSERVANT score						
	In-hospital					
	30d	4	683	13,617	0.597 (0.544–0.649)	71.4
	1 year	1	95	823	0.590 (0.535–0.645)	NA
	3 years	NA	NA	NA	NA	NA
	5 years	NA	NA	NA	NA	NA
	Overall	5	778	14,440	0.594 (0.554–0.633)	62.1
STS risk model						
	In-hospital	2	60	1,984	0.714 (0.588–0.840)	71
	30d	9	1,791	46,080	0.640 (0.602–0.677)	73.8
	1 year	1	3,230	20,704	0.630 (0.620–0.640)	NA
	3 years	NA	NA	NA	NA	NA
	5 years	1	18	620	0.715 (0.593–0.837)	NA
	Overall	13	5,099	69,388	0.648 (0.622–0.674)	70.2
ACC TAT risk model						
	In-hospital	1	14	1,038	0.738 (0.588–0.888)	NA
	30d	5	1,318	29,212	0.630 (0.616–0.645)	0
	1 year	NA	NA	NA	NA	NA
	3 years	NA	NA	NA	NA	NA
	5 years	NA	NA	NA	NA	NA
	Overall	6	1,332	30,250	0.632 (0.616–0.648)	14.2
New ML models						
	In-hospital	4	4,452	2,18,956	0.794 (0.787–0.800)	0
	30d	7	2,887	86,937	0.699 (0.658–0.739)	89.3
	1 year	9	638	5,257	0.785 (0.752–0.817)	80.3
	3 years	1	50	330	0.670 (0.595–0.745)	NA
	5 years	2	424	942	0.772 (0.743–0.801)	27.2
	Overall	23	8,451	3,12,422	0.757 (0.733–0.782)	95.4
Other scoring systems						
	Overall	32	9,095	1,53,606	0.675 (0.639–0.711)	97.7

New ML models—new machine learning models.

**Table 3:** SEN and SPE of various models.

Model	*n*	SEN (95% CI)	*I* ^2^	SPE (95% CI)	*I* ^2^
EuroSCORE I	6	0.57 (0.54–0.60)	77.51	0.59 (0.55–0.63)	97.45
EuroSCORE II	12	0.60 (0.57–0.62)	42.51	0.58 (0.54–0.62)	95.37
France II	3	NA	NA	NA	NA
OBSERVANT score	2	NA	NA	NA	NA
STS risk model	11	0.63 (0.57–0.68)	50.83	0.62 (0.57–0.67)	97.92
ACC TAT risk model	4	0.70 (0.57–0.80)	75.17	0.55 (0.48–0.62)	98.77
New ML models	9	0.66 (0.59–0.73)	75.49	0.77 (0.67–0.84)	95.35
Other scoring systems	21	0.64 (0.59–0.68)	87.28	0.66 (0.61–0.70)	99.77
Overall	71	0.61 (0.57–0.65)	93.21	0.61 (0.54–0.67)	99.86

### 3.6 Detailed results of subgroup analysis

Our study examined in-hospital, 30-day, 1-year, 3-year, and 5-year postoperative mortality. Our analysis showed that machine learning models were more accurate in predicting short-term mortality than long-term mortality. Furthermore, we identified that machine learning models were more accurate than traditional scoring systems.

## 4 Discussion

TAVR, although widely used, has a high mortality. The mortality of TAVR is 3.2% during hospitalization, 4.8% at 30 days, and 14% at one year. Therefore, early prediction of mortality after TAVR remains a research hot. In the existing studies, several traditional scoring systems have been validated, but their C-index for mortality is worrisome. Among them, EuroSCORE I had a C-index of 0.61 (95% CI: 0.588–0.632), SEN of 0.57 (95% CI: 0.54–0.60), and SPE of 0.59 (95% CI: 0.55–0.63). EuroSCORE II had a C-index of 0.615 (95% CI: 0.588–0.60), SEN of 0.60 (95% CI: 0.57–0.62), and SPE of 0.58 (95% CI: 0.54–0.62). The STS risk mode had a C-index of 0.648 (95% CI: 0.622–0.674), SEN of 0.63 (95% CI: 0.57–0.68), and SPE of 0.62 (95% CI: 0.57–0.67).

## 5 Traditional scoring systems

In our protocol registered on PROSPERO, we planned to explore the performance of machine learning for predicting mortality and summarize its predictive value. However, during our research, we found that there were still a large number of studies comparing traditional scoring systems. Therefore, we also compared the performance of these scoring systems with machine learning and further discussed whether machine learning had advantages over traditional scoring systems. Our study focused on EuroSCORE I, EuroSCORE III, France III, the OBSERVANT score, the STS risk model, the ACC TAT risk model, and other popular models for predicting mortality after TAVR. Among them, EuroSCORE I is an objective assessment system for predicting surgical or in-hospital mortality, based on a European cardiac surgery database. As a valid indicator of quality of care, surgical mortality needs to be associated with patient risk, and therefore, reliable risk classification models are required (47). Nonetheless, EuroSCORE I now overpredicts risk because cardiac surgery outcomes have substantially improved with technological advances, reducing mortality rates. Thus, the model may now be inappropriate for current cardiac surgery (10, 39).

EuroSCORE II is developed based on EuroSCORE I. EuroSCORE II reassesses the effect of predictors. Age remains a significant predictor of mortality after the age of 60 years, but its effect is reduced compared with the previous one. Symptomatic status is associated with an increased risk, and only CCS grade 4 angina is associated with a poor prognosis, whereas a higher NYHA class is associated with a higher risk. Therefore, NYHA classes II, III, and IV, but only angina CCS class 4, are included in the model. BMI is weakly associated with mortality. Low BMI appears to increase the risk of hospital death, but high BMI does not. Diabetes mellitus is considered. Hepatic failure is associated with an increase in cardiac surgical mortality, but this risk factor is not usually represented in risk models. Therefore, the serum albumin concentration is the least affected by cardiac treatment and is the most objective and widely available test. However, there is no relationship between serum albumin and mortality risk (48).

At the same time, the American College of Thoracic Surgeons has developed the STS score, a scoring system based on a US national database. The biggest difference in the measurement of risk factors between EuroSCORE II and the STS score is that the STS score includes race as a determinant in predicting mortality (49).

## 6 Emerging predictors

According to our study, none of the existing prediction models is able to predict mortality reliably. Therefore, new models are required. The following factors can be included in prediction models: physical fitness, vulnerability assessment, right heart function, pulmonary circulatory load, and indicators of vascular inflammation and stress. A large body of clinical evidence shows that traditional cardiorespiratory fitness indicators alone do not fully reflect the functional reserve and recovery ability of older patients. In contrast, vulnerability scores, which comprise walking speed, grip strength, and poor body mass index, can better capture the overall functional status of patients and play an important role in early prognostic assessment (50). Furthermore, right ventricular dysfunction or pulmonary hypertension may lead to persistent cardiac insufficiency and circulatory mechanical abnormalities after TAVR, and its impact on early mortality risk is progressively being emphasized (51). Additionally, some studies have included biomarkers such as white blood cell counts and C-reactive protein to reflect the impact of systemic inflammation and stress on the risk of adverse events after valve intervention (52). Besides, the gender factor has been increasingly explored in the last two years. Women have a higher all-cause mortality rate than men, but the odds of needing a pacemaker and acute kidney injury are significantly lower in female patients (53). Previous AF and new-onset AF have also been found to be associated with 30-day mortality, stroke occurrence, and length of hospital stay after TAVR (54). These emerging predictors are in the models because they often complement the traditional scoring systems by omitting important information about patients receiving TAVR, and improve the predictive applicability of the models in different populations or specific subgroups. Excessive LV volume and LV fibrosis are associated with poor prognosis in patients with severe AS treated with TAVR, and this factor could be considered as a predictor (55).

In addition to the scoring tools analyzed in our study, other scores for the prediction of mortality after TAVR should also be considered. For instance, the Emory Risk Score appears to have relatively high performance for predicting the risk of permanent pacemaker implantation (PPI) [31699374]. Some studies suggest a significant association between PPI and mortality risk [30019825, 35837611, 35138367]. Therefore, the potential predictive value of the Emory Risk Score for mortality risk should be further explored. We note that the Emory Risk Score and the mortality risk scores (i.e., EuroSCORE II and STS-PROM) focus on different predictive domains, with minimal overlap in predictors. The Emory Risk Score focuses on conduction system vulnerability and procedural factors, while the mortality risk scores capture systemic comorbidity burden and end-organ function. Therefore, the Emory Risk Score may be less likely to directly confound the c-statistic or calibration of mortality prediction models at the population level. Nevertheless, future research still needs to explore the specific performance of the Emory Risk Score and similar prediction tools for PPI in predicting the risk of mortality.

## 7 Clinical implications

A recent clinical trial has found that at 30 days after TAVR, significant reductions in left ventricular (LV) global work index (GWI), global constructive work (GCW), and global wasted work (GWW) are observed; only patients without LV dyssynchrony show an increase in global work efficiency (GWE); and echocardiographic calculations of the MW index are accurate in patients with severe AS (56). This shows the role of TAVR in the ventricular structure of patients, and TAVR is important for prolonging the life of patients and improving their quality of life.

For the traditional prediction models, predictors need to be updated. In contrast, machine learning does not have this shortcoming. Once trained based on historical data, machine learning can make accurate predictions, because a machine learning algorithm can learn through feedback from an objective or outcome set in the training data to constantly improve the performance of machine learning models. Thus, machine learning has a unique advantage in prognostic prediction for TAVR patients.

### 7.1 Strengths and limitations of this study

As far as we know, our study is the first meta-analysis of models for predicting the risk of mortality after TAVR. However, this meta-analysis has some limitations. Firstly, our study includes several scoring systems (models), but due to the limited number of included studies, only one study validates other systems. Therefore, we do not discuss it in detail. Secondly, only a very few included studies investigate new machine learning models, which are constructed based on different risk factors and methods. Thus, they cannot be further discussed. Thirdly, we have validated each scoring system and discussed its performance in predicting the risk of mortality at different time points after TAVR. However, only a very few studies report long-term follow-up, especially 3-year mortality. Therefore, our results need to be interpreted with caution. Fourthly, for the performance of predictive models, a large number of studies only focus on c-index or SEN and SPE, while reports on model calibration and clinical applicability are very limited. Therefore, we are unable to analyze these parameters. This limitation is prevalent in current predictive model research in this field. Future research should further improve the evaluation indicators to objectively evaluate model performance. Fifthly, factors such as age, frailty, comorbidities, and other cardiac interventions have a potential impact on the predictive value of models for mortality. However, our included studies rarely describe factors such as frailty, comorbidities, and other cardiac interventions. Therefore, we are unable to further discuss the potential impact of these factors on the predictive value of the models. In addition, the age range in the original studies is too wide, and subgroup analysis by age is not performed. Therefore, this study also does not further discuss its potential impact, although it is a very important predictor in newly constructed machine learning models.

## 8 Conclusion

Predicting the risk of mortality after TAVR with currently available mainstream scoring tools remains challenging. Our study shows that the STS risk model has the highest C-index for predicting the risk of mortality after TAVR, but it needs to be further improved. Therefore, subsequent studies should explore more effective prediction models or more accurate predictors to update currently available scoring tools.

## Data Availability

The original contributions presented in the study are included in the article/Supplementary Material, further inquiries can be directed to the corresponding author/s.
