# Supplementary material for: Early predictive value of prediction models for mortality after transcatheter aortic valve replacement: a systematic review and meta-analysis
Source: Front Cardiovasc Med. 2026 Jul 15;13:1757852. doi: 10.3389/fcvm.2026.1757852 (PMC13415785; doi:10.3389/fcvm.2026.1757852)
Supplement: Supplementary file 1 [file Datasheet1.docx]

# Supplementary Material 1 Literature search strategy

**1.Pubmed**

| Search number | Query |
| --- | --- |
| #1 | ("Transcatheter Aortic Valve Replacement"[Mesh] OR "Transcatheter Aortic Valve Implantation"[Mesh] OR TAVR[tiab] OR TAVI[tiab] OR "transcatheter aortic valve"[tiab]) |
| #2 | ("Mortality"[Mesh] OR "Death"[Mesh] OR mortality[tiab] OR death[tiab] OR survival[tiab] OR outcome*[tiab] OR prognosis[tiab] OR prognostic[tiab]) |
| #3 | ("Risk Assessment"[Mesh] OR "Risk Factors"[Mesh] OR "Models, Statistical"[Mesh] OR "Machine Learning"[Mesh] OR "Algorithms"[Mesh] OR risk*[tiab] OR predict*[tiab] OR model*[tiab] OR score*[tiab] OR algorithm*[tiab] OR index[tiab] OR indices[tiab] OR "risk score"[tiab] OR "prediction model"[tiab]) |
| #4 | #1 AND #2 AND #3 |

**2.Cochrane**

| Search number | Query |
| --- | --- |
| #1 | ((MH "Heart Valve Prosthesis Implantation") OR ("transcatheter aortic valve replacement" OR "transcatheter aortic valve implantation" OR TAVR OR TAVI OR "transcatheter aortic valve")) |
| #2 | (MH "Mortality") OR (MH "Death") OR mortality OR death OR survival OR outcome OR prognosis OR prognostic) |
| #3 | (MH "Risk Assessment") OR (MH "Risk Factors") OR (MH "Models, Statistical") OR (MH "Algorithms") OR "risk score" OR "prediction model" OR "prognostic model" OR risk OR predict OR model OR score OR algorithm OR index OR indices) |
| #4 | #1 AND #2 AND #3 |

**3.Embase**

| Search number | Query |
| --- | --- |
| #1 | ('transcatheter aortic valve replacement'/exp OR 'transcatheter aortic valve implantation'/exp OR  'tavr':ab,ti OR 'tavi':ab,ti OR 'transcatheter aortic valve':ab,ti) |
| #2 | ('mortality'/exp OR 'death'/exp OR 'mortality':ab,ti OR 'death':ab,ti OR 'survival':ab,ti OR 'outcome':ab,ti OR 'prognosis':ab,ti OR 'prognostic':ab,ti) |
| #3 | ('risk assessment'/exp OR 'risk factor'/exp OR 'statistical model'/exp OR 'machine learning'/exp OR 'algorithm'/exp OR 'risk score':ab,ti OR 'prediction model':ab,ti OR 'prognostic model':ab,ti OR 'risk':ab,ti OR 'predict':ab,ti OR 'model':ab,ti OR 'score':ab,ti OR 'algorithm':ab,ti OR 'index':ab,ti OR 'indices':ab,ti) |
| #4 | #1 AND #2 AND #3 |

**4.Web of science**

| Search number | Query |
| --- | --- |
| #1 | TS=("transcatheter aortic valve replacement" OR "transcatheter aortic valve implantation" OR TAVR OR TAVI OR "transcatheter aortic valve") |
| #2 | TS=(mortality OR death OR survival OR outcome* OR prognosis OR prognostic) |
| #3 | TS=("risk score" OR "prediction model" OR "prognostic model" OR risk OR predict OR model OR score OR algorithm OR index OR indices) |
| #4 | #1 AND #2 AND #3 |


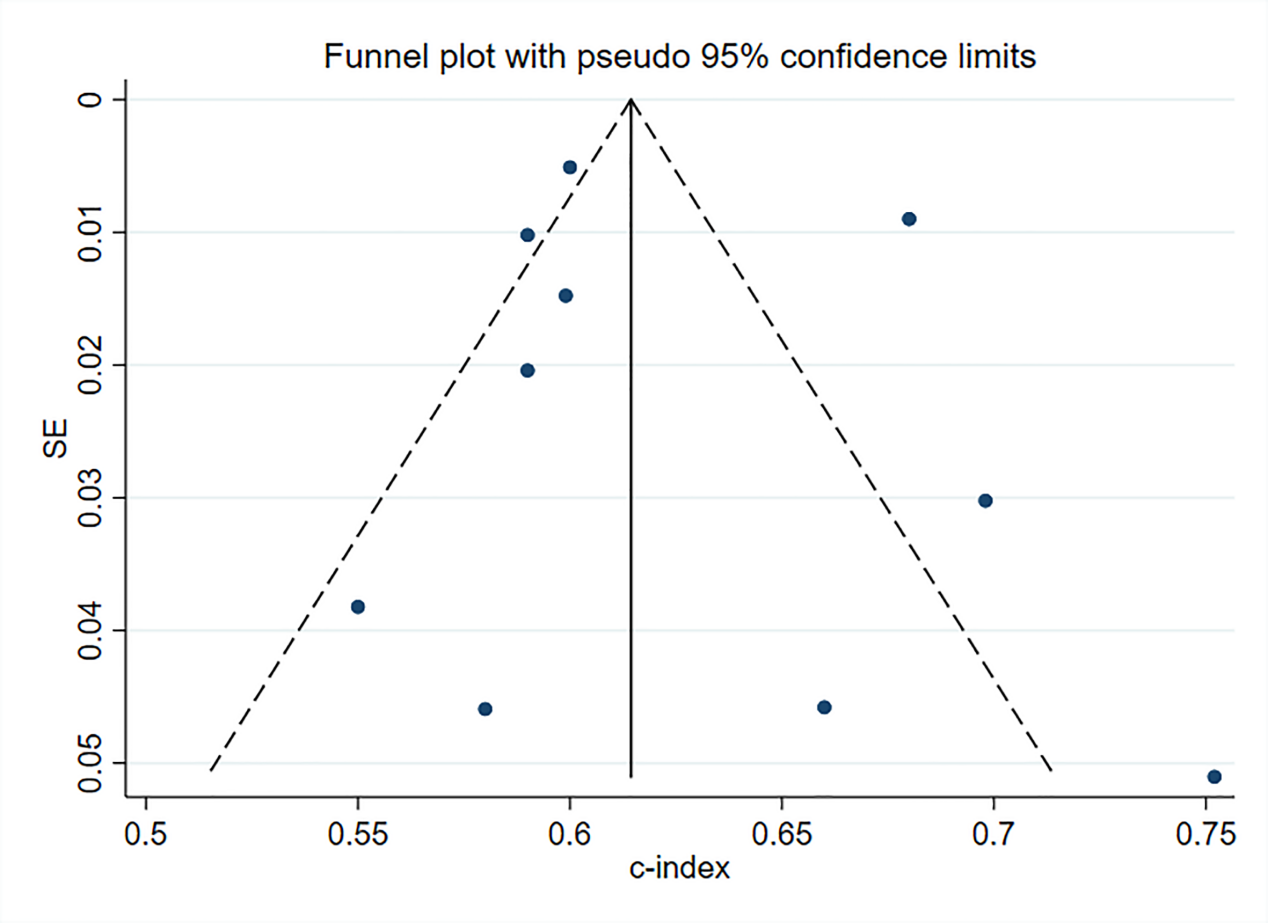


Figure S1 Forest plot for meta-analysis of mortality predicted by EuroSCORE I


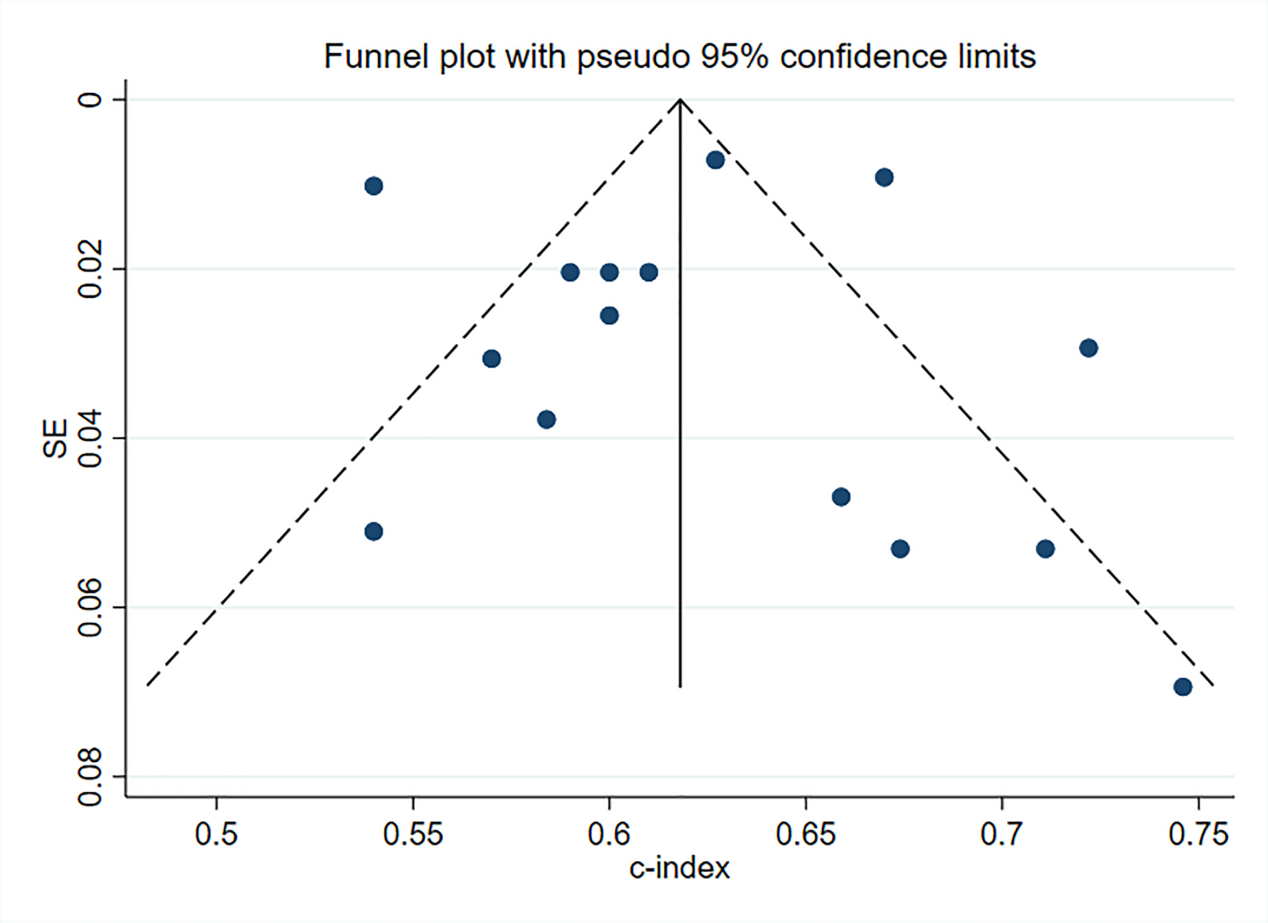


Figure S2 Forest plot for meta-analysis of mortality predicted by EuroSCORE II


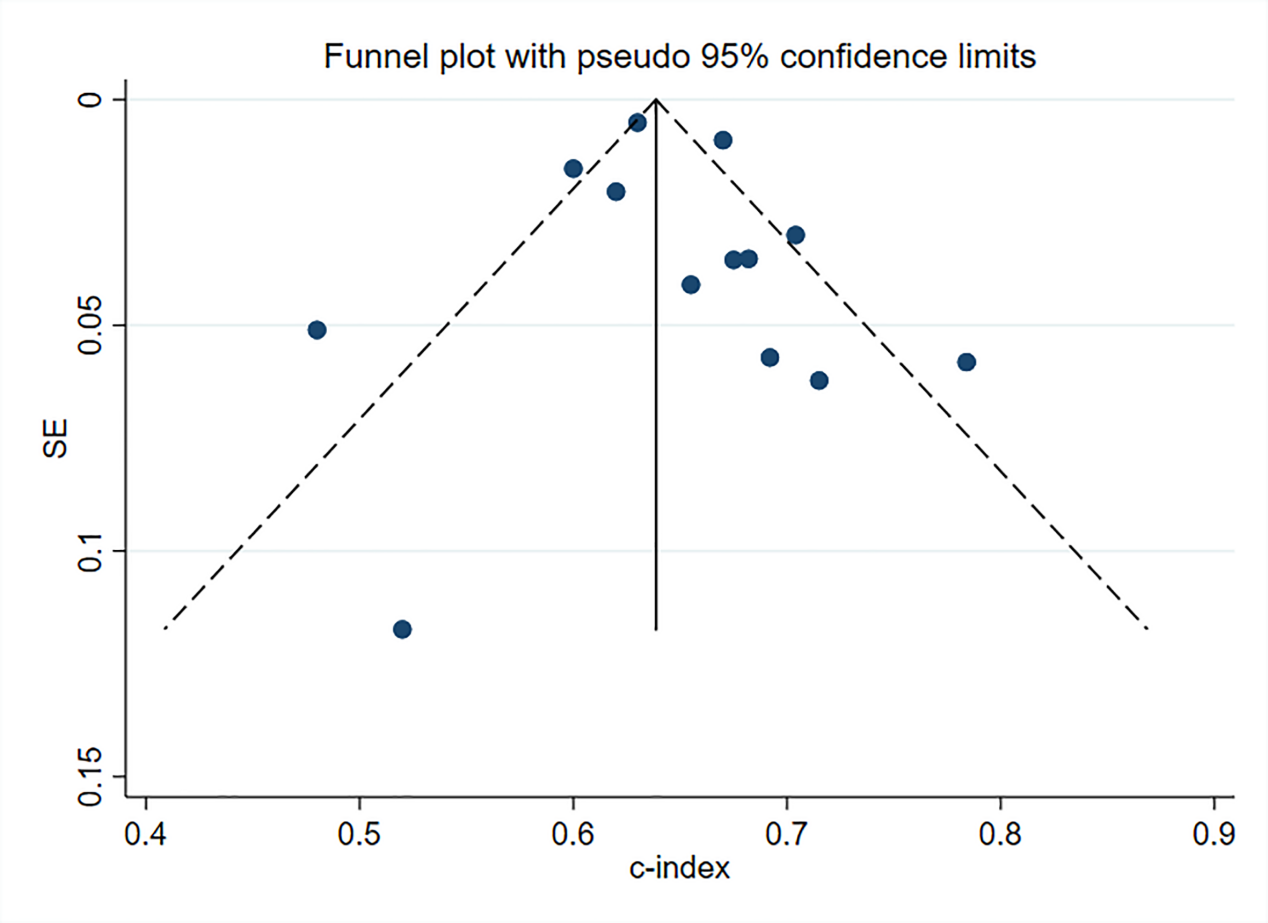


Figure S3 Forest plot for meta-analysis of mortality predicted by the STS risk model


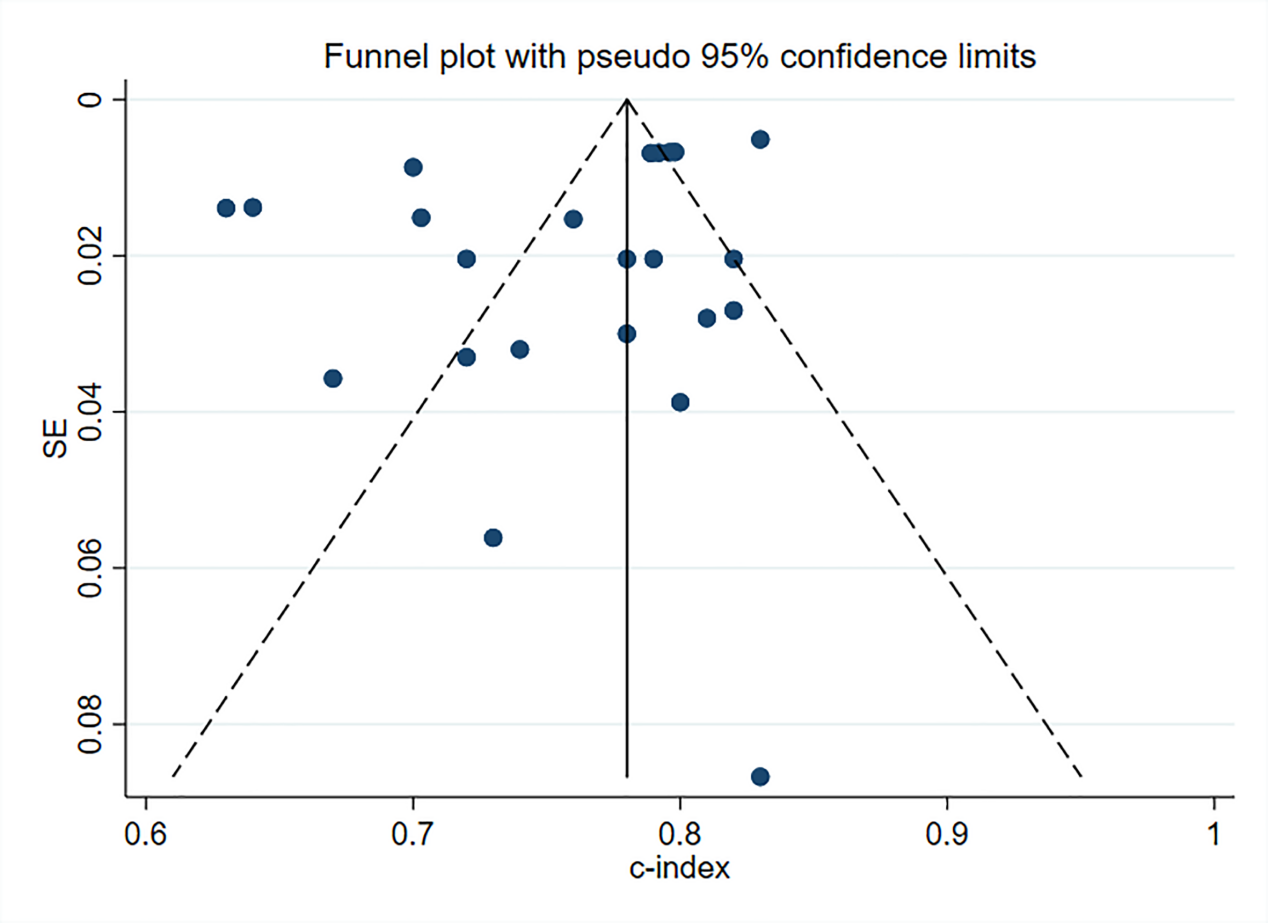


Figure S4 Forest plot for meta-analysis of mortality predicted by new ML models
